# Formate cross‐feeding and cooperative metabolic interactions revealed by transcriptomics in co‐cultures of acetogenic and amylolytic human colonic bacteria

**DOI:** 10.1111/1462-2920.14454

**Published:** 2018-11-22

**Authors:** Jenny A. Laverde Gomez, Indrani Mukhopadhya, Sylvia H. Duncan, Petra Louis, Sophie Shaw, Elaina Collie‐Duguid, Emmanuelle Crost, Nathalie Juge, Harry J. Flint

**Affiliations:** ^1^ Gut Health Group The Rowett Institute, University of Aberdeen Aberdeen UK; ^2^ Centre for Genome Enabled Biology and Medicine University of Aberdeen Old Aberdeen UK; ^3^ The Gut Health and Food Safety Institute Strategic Programme Quadram Institute Bioscience Norwich UK

## Abstract

Interspecies cross‐feeding is a fundamental factor in anaerobic microbial communities. In the human colon, formate is produced by many bacterial species but is normally detected only at low concentrations. *Ruminococcus bromii* produces formate, ethanol and acetate in approximately equal molar proportions in pure culture on RUM‐RS medium with 0.2% Novelose resistant starch (RS3) as energy source. Batch co‐culturing on starch with the acetogen *Blautia hydrogenotrophica* however led to the disappearance of formate and increased levels of acetate, which is proposed to occur through the routing of formate via the Wood Ljungdahl pathway of *B. hydrogenotrophica*. We investigated these inter‐species interactions further using RNAseq to examine gene expression in continuous co‐cultures of *R. bromii* and *B. hydrogenotrophica*. Transcriptome analysis revealed upregulation of *B. hydrogenotrophica* genes involved in the Wood‐Ljungdahl pathway and of a 10 gene cluster responsible for increased branched chain amino acid fermentation in the co‐cultures. Cross‐feeding between formate‐producing species and acetogens may be a significant factor in short chain fatty acid formation in the colon contributing to high rates of acetate production. Transcriptome analysis also indicated competition for the vitamin thiamine and downregulation of dissimilatory sulfate reduction and key redox proteins in *R. bromii* in the co‐cultures, thus demonstrating the wide‐ranging consequences of inter‐species interactions in this model system.

## Introduction

The anaerobic microbial communities found in the mammalian large intestine and rumen represent the most densely‐colonized microbial ecosystems in nature (Whitman *et al*., 1998). Metabolite cross‐feeding is recognized as a key feature of such systems that contributes to their complexity, stability and productivity (Sieber *et al*., 2012; Costliow and Degnan, 2017). Of particular importance are cross‐feeding interactions that can influence the metabolic pathways involved in fermentative metabolism and hence the energetics of bacterial metabolism. One of the first examples of this was provided by the rumen bacterium *Ruminococcus albus*; when grown on a carbohydrate energy source, *R. albus* derives ATP via acetate production from acetyl‐CoA, but has to route acetyl‐CoA partly to ethanol as a hydrogen sink. In the presence of a H_2_ ‐utilizing methanogen, however, *R. albus* is able to divert more carbon to acetate, and thus gains additional ATP (Wolin *et al*., 1997).

Recent work on the dominant *Ruminococcus* species found in the human colon, *R. bromii*, has shown that this organism has an important role in the degradation of dietary resistant starch (Walker *et al*., 2011; Ze *et al*., 2012). Rather little is known about *R. bromii* metabolism, although this bacterium is reported to have limited ability to synthesize required vitamins (Ze *et al*., 2012; Magnúsdóttir *et al*., 2015; Mukhopadhya *et al*., 2018). *R. bromii* is reported to produce ethanol, acetate and formate in pure culture with fructose as the carbon source (Herbeck and Bryant, 1974), suggesting that excess reducing equivalents are disposed largely through the production of formate and ethanol in this species. Methanogenic archaea are reported to utilize either formate or H_2_ and CO_2_ and have been shown to influence metabolism in hydrogen‐producing Firmicutes (Chassard and Bernalier‐Donadille, 2005) but are present in very low numbers in around 50% of the human population (Florin *et al*., 2000). On the other hand, another group of H_2_ and/or formate‐utilizing microorganisms, acetogenic bacteria, appear to be abundant and widespread in all healthy individuals (Doré *et al*., 1995; Rey *et al*., 2010). We decided therefore to explore the potential for such interactions using the acetogenic human colonic bacterium *Blautia hydrogenotrophica* and *R. bromii* as a model system. Co‐culture is shown to have wide‐ranging consequences for metabolic outputs and for gene expression in both species through interactions that involve vitamins, redox pathways, alternative energy sources and carbon flow.

## Results and discussion

### *Co‐cultivation of* R. bromii *with* B. hydrogenotrophica *in batch culture*

**Figure 1:**
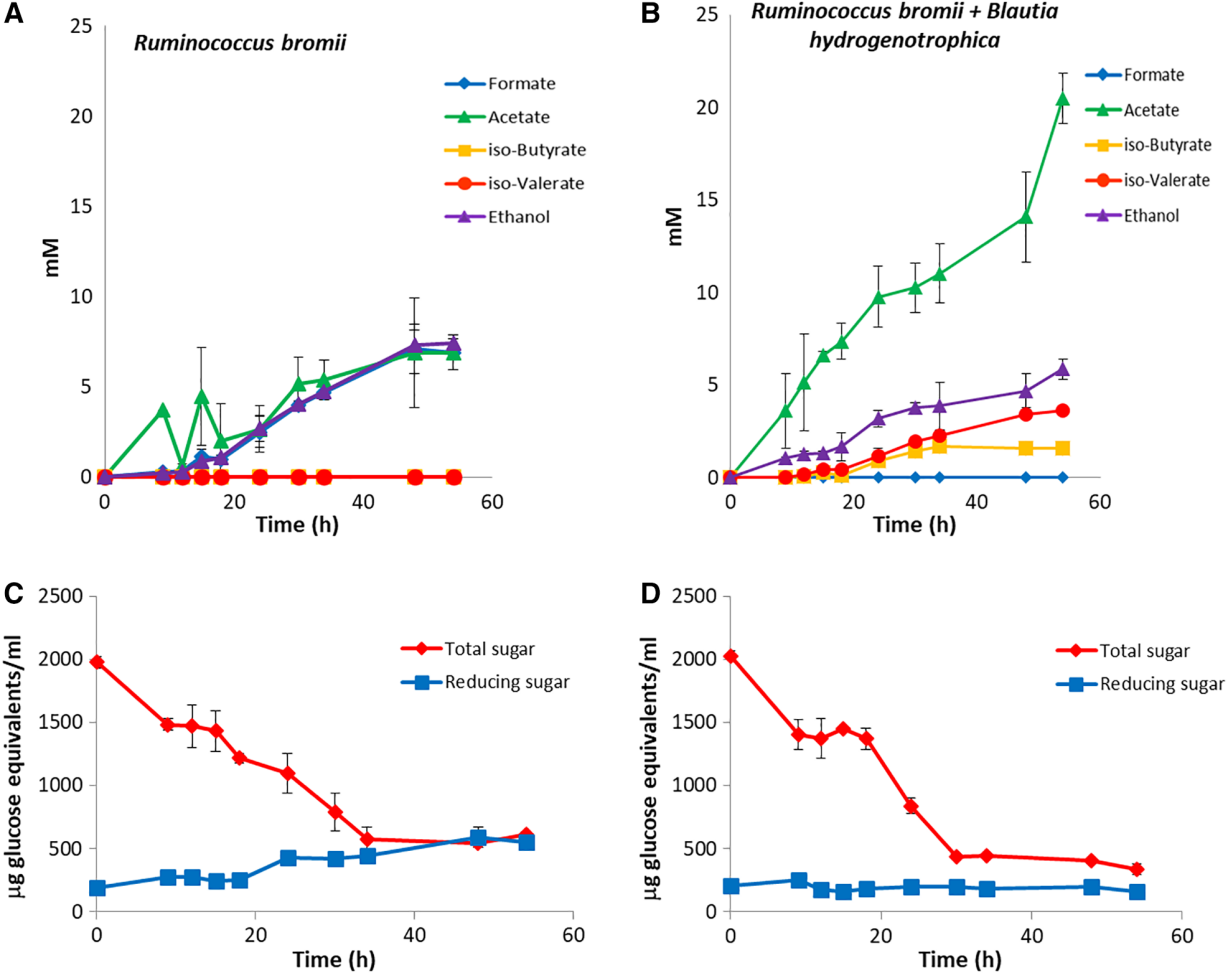
Fermentation products (A, B) and substrate utilization (C, D) for *Ruminococcus bromii* monocultures (A, C) and for co‐cultures with *B. hydrogenotrophica* grown in batch culture in RUM‐RS medium (B, D). Results are shown as the means (with standard deviations) for triplicate cultures that were inoculated with 100 μl of overnight cultures simultaneously with the two bacteria at time 0. Cultures were sampled aseptically under anaerobic conditions at the time points shown (see Methods). In (C) and (D) reducing sugar refers to the culture supernatant only, whereas total sugar refers to the whole culture. Reducing sugar standard deviation values were small and hence are not visible on the figure.

The major fermentation products of *R. bromii* L2‐63 when grown in batch culture on RUM‐RS medium with 0.2% Novelose resistant starch (RS3) as energy source were acetate, formate and ethanol (Fig. 1A)**.** *R. bromii* utilized at least 70% of the RS3 substrate, based on the change in total hexose in the culture, although free reducing sugar accumulated during the incubation as reported previously (Fig. 1C) (Ze *et al*., 2012)**.** The acetogenic bacterium *B. hydogenotrophica* DSM 10507 could not utilize starch, but grew on RUM‐G medium containing 0.2% glucose, producing acetate together with ethanol, formate and lactate. Growth of *B. hydrogenotrophica* on RUM‐G was found to be stimulated by the presence of formate, with the production of additional acetate (Supporting Information Fig. S1). Formate is assumed to be assimilated via the methyl branch of the Wood‐Ljungdahl pathway, as has been demonstrated for *Bryantella formatexigenes* (Wolin *et al*., 2003). *B. hydrogenotrophica* failed to grow in RUM medium in the absence of added glucose with or without added formate (Supporting Information Fig. S1).

Co‐cultivation of *R. bromii* and *B. hydrogenotrophica* in batch culture with resistant starch as the energy source (RUM‐RS) resulted in a major increase in the yield of acetate when compared with the *R. bromii* monoculture, while no formate was detected in co‐culture (Fig. 1B). The gradual increase in soluble reducing sugar that was seen in *R. bromii* monocultures was absent in co‐culture (Fig. 1C and D) suggesting that *B. hydrogenotrophica* was able to utilize sugars released from RS by *R. bromii* **.** In addition, the co‐culture gave rise to the branched chain fatty acids iso‐butyrate and iso‐valerate at concentrations not seen in the *R. bromii* monoculture on starch or in *B. hydrogenotrophica* monoculture on glucose (Fig. 1A and B). This indicates that there was an increase in fermentation of branched chain amino acids by one of the two species in the co‐culture. *B. hydrogenotrophica* can form the branched chain fatty acids, iso‐butyrate and iso‐valerate from branched chain amino acids (Rasmussen *et al*., 1988), in addition to the major fermentation products acetate, lactate and ethanol.

### *Interactions between* R. bromii *and* B. hydrogenotrophica *in continuous culture*

**Figure 2:**
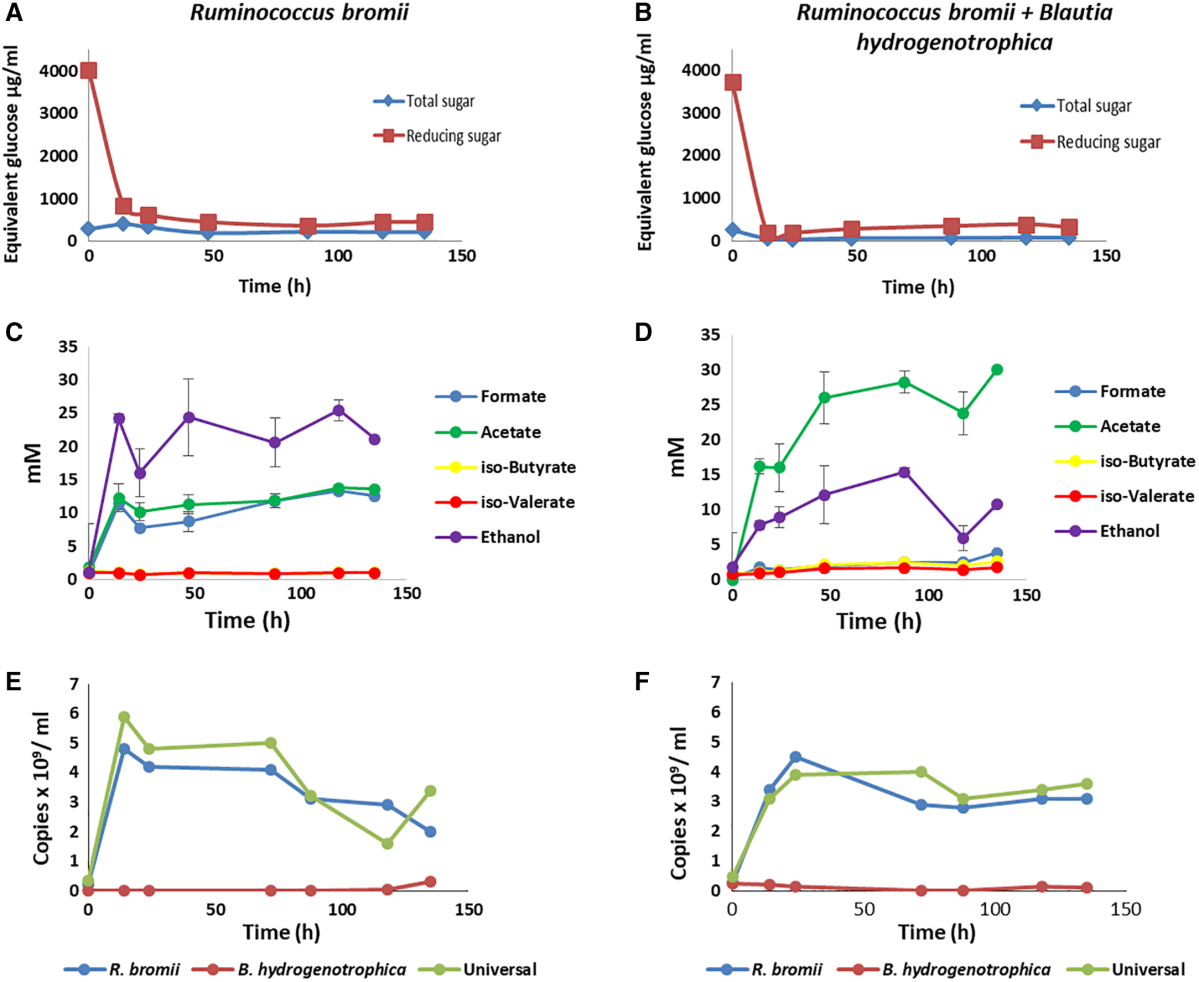
Interactions of *R. bromii* and *B. hydrogenotrophica* in continuous culture in RUM‐S medium containing 0.5% soluble starch. Fermentors inoculated with *R. bromii* only and with *R. bromii* plus *B. hydrogenotrophica* were run in parallel. Substrate utilization (A, B), fermentation products (C, D) and bacterial populations (E, F) estimated by 16S rRNA gene‐targeted qPCR are shown for the mono‐culture (A, C, E) and co‐culture (B, D, F) respectively, based on single samples taken at each time point. Results shown here correspond to fermentors R2 and C2 (Supporting Information Figs S2 and S3); results from a second experiment (R1, C1) and for *B. hydrogenotrophica* monocultures grown in RUM‐G medium (with glucose as energy source) (B1, B2) are also shown in Supporting Information Figs S2 and S3 for comparison. Reducing and total sugar standard deviation values were small (ranging from 0.076 to 22.79 μg ml^−1^ for reducing sugar and 7.39 to 153.59 μg ml^−1^ for total sugar) and hence the error bars are not visible on the figure. For the qPCR data, each value plotted is based on mean of duplicate Cq values.

We decided to employ anaerobic continuous culture in order to explore these interactions further. It proved possible to grow *R. bromii* in continuous culture by supplying RUM‐S medium containing soluble starch as sole added energy source, although it was found necessary to renew the supply medium daily because of the lability of certain added vitamins (see Materials and Methods). Mean product concentrations for continuous monocultures of *R. bromii* grown with input of 0.5% starch were acetate 11.3 mM, ethanol 18.7 mM and formate 10.7 mM (means of 11 time points from two experiments – Fig. 2, Supporting Information Figs S2 and S3). This corresponds to an approximate ratio 1:2:1, as compared to a molar ratio closer to 1:1:1 in batch culture (Fig. 1).

*B. hydrogenotrophica* was able to grow in continuous culture in RUM‐G medium with 0.2% glucose as energy source, producing acetate as the main fermentation acid. Supply of 0.5% glucose resulted in formation of lactate and ethanol in addition to acetate but did not increase cell numbers (Supporting Information Figs S2 and S3).

Continuous co‐cultures of *R. bromii* with *B. hydrogenotrophica* supplied with RUM‐S medium supported a dominant population of *R. bromii* together with a much smaller population (by a factor of log_2_ 2–4 fold) of *B. hydogenotrophica* as assessed by qPCR analysis (Fig. 2E and F). The *R. bromii* 16S rRNA gene copy number did not change noticeably in the co‐culture relative to the monoculture. Co‐cultures gave rise to increased acetate (23.1 mM) and decreased ethanol (12.9 mM) and formate (2.2 mM) concentrations (means of 9 time points from two experiments) when compared with the *R. bromii* monoculture. In addition, concentrations of branched chain fatty acids (BCFA) were increased in co‐culture relative to monocultures of *R. bromii* on RUM‐S (iso‐butyrate 1.9 cf. 1.0; iso‐valerate 1.8 cf. 0.9) (Fig. 2C and D). The low concentrations of reducing sugar that were maintained in the *R. bromii* monoculture were further decreased in the co‐culture, indicating utilization by *B. hydrogenotrophica* (Fig. 2A and B).

**Figure 3:**
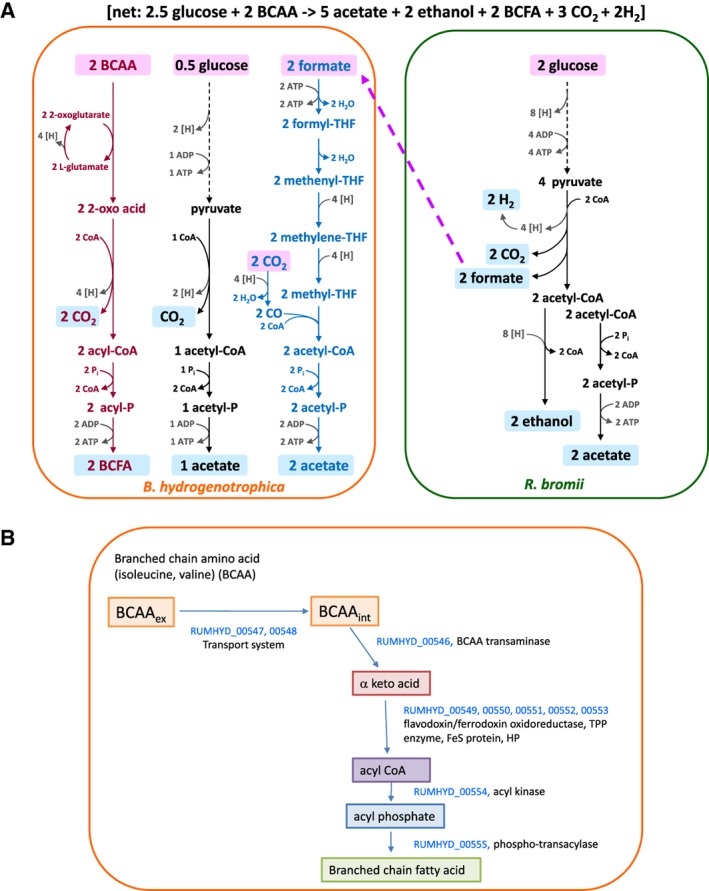
A. Scheme for proposed metabolic interactions between *Ruminococcus bromii* and *Blautia hydrogenotrophica*. This scheme approximates the fermentation stoichiometries observed in co‐cultures between *R. bromii* and *B. hydrogenotrophica* in batch culture on RUM‐RS medium. The relative amounts of glucose and branched chain amino acids fermented by *B. hydrogenotrophica* are not known precisely, but the assumption made here approximates to observed yield of BCFA in the co‐culture (see Fig. 1). B. Genes proposed to be involved in branched chain amino acid fermentation in *Blautia hydrogenotrophica*.

A proposed explanation for the metabolic shifts seen in co‐cultures is provided by the metabolic scheme in Fig. 3A. When grown in monoculture, ethanol production by *R. bromii* provides a sink for reducing equivalents (NADH) from glycolysis and acetate production from acetyl‐CoA is limited to approximately one mol acetate per mol glucose fermented, while production of formate reduces the formation of gaseous hydrogen. In the co‐culture, *B. hydrogenotrophica* may be able to benefit from some glucose released from starch by *R. bromii* (Ze *et al*., 2012) (Fig. 1). In addition, however, based on evidence discussed further below, *B. hydrogenotrophica* appears able to utilize branched chain amino acids as energy sources in the co‐culture (Fig. 3B). We assume that formate produced by *R. bromii* in the co‐culture is routed into additional acetate via the Wood‐Ljungdahl pathway in *B. hydrogenotrophica* **.** The relatively low cell numbers achieved by *B. hydrogenotrophica* in the co‐culture were nevertheless sufficient to consume all of the formate produced by *R. bromii* and to greatly increase the yield of acetate. The metabolic scheme suggested in Fig. 3A predicts the formation of approximately 2 mol acetate and 1 mol ethanol per mol glucose consumed by the co‐cultures, with branched chain amino acid fermentation by *B. hydrogenotrophica* contributing additional ATP and NADH, with the formation of BCFA. Approximate carbon balances are shown in Supporting Information Table S1. An additional possibility, not shown in Fig. 3A, is that H_2_ is transferred from *R. bromii* to *B. hydrogenotrophica* in the co‐culture. Indeed, we do not rule out the possibility that *R. bromii* metabolism is modified in the co‐culture to produce more hydrogen and less formate.

### *Transcriptome analysis*

To determine the molecular pathways underpinning the observed shifts in fermentation products, transcriptomics analyses were carried out using continuous culture experiments under conditions that approximate to a steady state. Briefly, mRNA was extracted from a total of nine samples, three samples from co‐cultures and three samples from monocultures of each species (Supporting Information Fig. S3), and gene expression was measured by RNA‐seq as described. Gene expression data were analysed using the draft genomes of the two bacteria as a reference and the ratio of expression in the co‐culture relative to that in the monoculture was determined for transcripts of each species. A total of 235 million reads were produced from the nine samples, with an average of 26 million reads per sample (Supporting Information Table S2).

**Figure 4:**
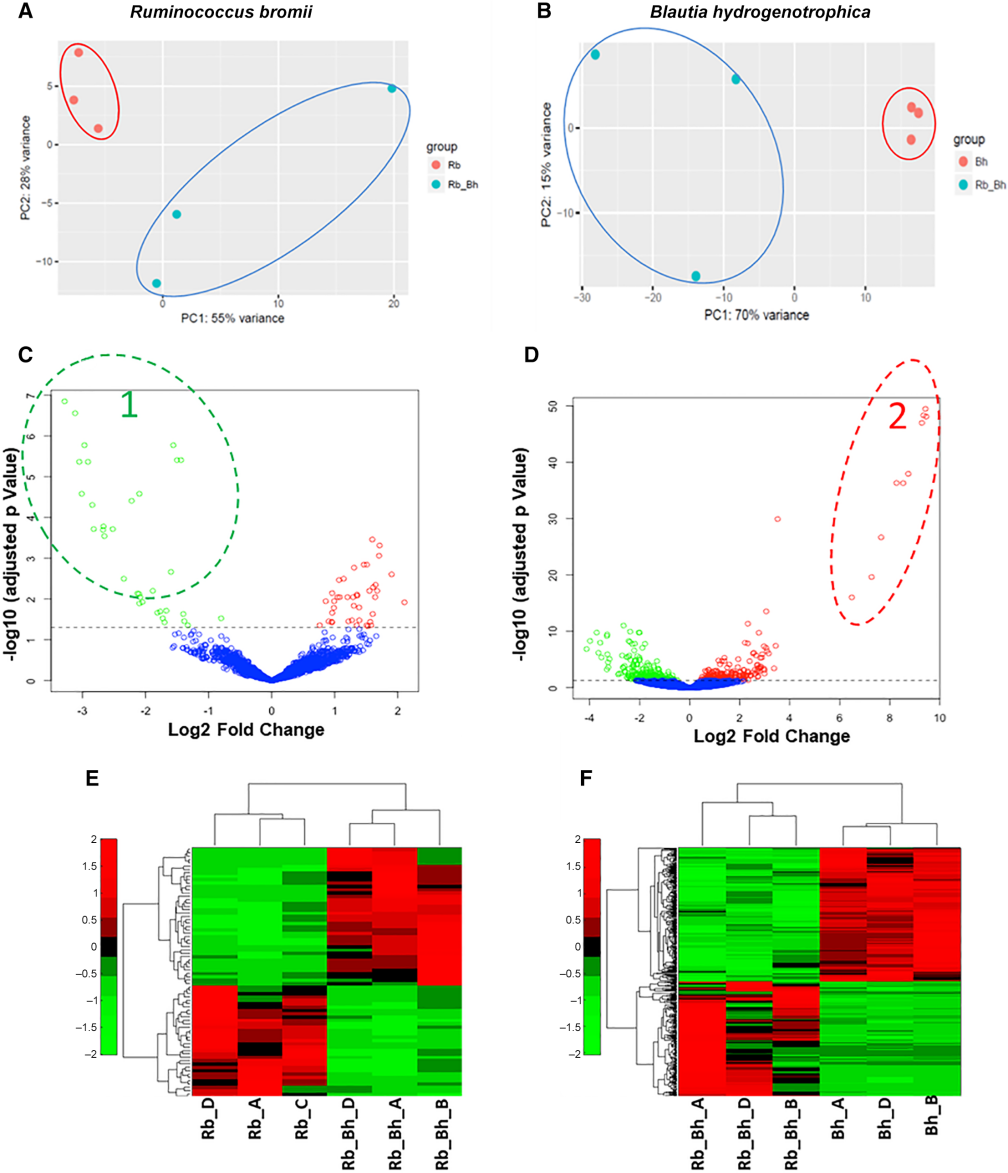
Principal‐component analysis, volcano plot and hierarchical cluster analysis of the RNA‐Seq samples examined in this study. A and B. Principal‐component analysis of the RNA‐seq reads for the *R. bromii* and *B. hydrogenotrophica* monoculture and co‐culture experiments, comparing all RNA‐Seq samples analysed. Red circles show monoculture, whereas blue circle shows co‐culture samples. (A) *R. bromii* and (B) *B. hydrogenotrophica*. (c and d) Volcano plots showing fold change and levels of significance for differential expression for all genes. The up and down regulated genes (based on an adjusted *p* < 0.05) are shown in red and green respectively and the genes with no change are shown in blue. C. *R. bromii* and (D) *B. hydrogenotrophica*. E and F. Heat maps showing expression of differentially expressed genes in *R. bromii* and *B. hydrogenotrophica*. Red shows high expression and green shows low expression. (E) *R. bromii* and (F) *B. hydrogenotrophica*.

Principal‐component analysis (PCA) of the normalized read counts (Fig. 4A and B) showed that samples from the monocultures clustered closely, indicating good reproducibility of gene expression between independent replicates. Samples from the co‐cultures showed greater variance but clearly grouped separately from the monocultures. A volcano plot (Fig. 4C and D) shows fold changes and levels of significance for differential expression for all genes, with highly dysregulated genes appearing farther to the left and right sides and highly significant changes appearing higher on the plot. A total of 75 *R. bromii* genes and 420 *B. hydrogenotrophica* genes were significantly differentially expressed (FDR ≤ 0.05) in the co‐culture compared to the respective monocultures (Fig. 4C and F). For *R. bromii*, gene expression changes ranged from −3 to 2 log_2_ fold change, whereas in *B. hydrogenotrophica* larger changes in gene expression were found (ranged from −4 to 9 log_2_ fold change; Fig. 4C and D). Hierarchical cluster analysis of the differentially expressed genes (Fig. 4E and F) support the PCA analysis, with co‐culture or monoculture samples clustering together.

### *Changes in the* R. bromii *transcriptome in continuous co‐culture with* B. hydrogenotrophica

Out of 75 *R. bromii* genes that showed differences in expression in the co‐culture relative to the monoculture (accounting for 3.5% of total *R. bromii* transcripts annotated in the reference genome used) 32 were upregulated and 43 were downregulated in the co‐culture. Seven differentially expressed genes were hypothetical proteins with no annotation matches (Supporting Information Table S3). Twenty *R. bromii* transcripts downregulated by co‐culture were present in a single cluster of linked genes (L2‐63_01134 – L2‐63_01158) consisting mainly of genes involved in sulfur and porphyrin metabolism (Table 1). These include a putative transcriptional regulator iscR, and genes coding for sulfate transport, sulfite reductase, thioredoxin and siroheme synthesis. This suggests that *R. bromii* shares with some other obligatory anaerobic bacteria the ability to reduce sulfate by the energy producing dissimilatory pathway in which sulfate (or sulfur) is the terminal electron acceptor (Wagner *et al*., 1998). This pathway starts with activation of sulfate by ATP to form adenylyl sulfate (APS), which is directly reduced to sulfite and then to sulfide by the dissimilatory sulfite reductase. The downregulation of the *R. bromii* sulfate reduction genes suggests that the activity of the acetogen, *B. hydrogenotrophica* in the co‐culture may provide an alternative electronic sink, thereby reducing the need for *R. bromii* to dispose of electrons by reducing sulfite, which may also benefit *R. bromii* energetically since sulfate reduction is ATP‐dependent (Rückert, 2016).

**Table 1:** Two gene clusters that showed the greatest transcriptional responses to co‐culture.

Gene	Log_2_ fold change	Annotation/likely function
**1. Clustered genes concerned with sulfur metabolism and siroheme synthesis in *Ruminococcus bromii*, downregulated when in co‐culture with *Blautia hydrogenotrophica***		
L2‐63_01134	−2.82	sbp Sulfate starvation induced protein
L2‐63_01135	−3.01	cysW_1 Sulfate transport system permease protein
L2‐63_01136	−3.29	cysW_2 Sulfate transport system permease protein
L2‐63_01137	−3.12	cysA Sulfate/thiosulfate import ATP‐binding protein
L2‐63_01140	−1.81	hemA Glutamyl‐tRNA reductase
L2‐63_01141	−1.7	cysG_1 Siroheme synthase, precorrin‐2 dehydrogenase
L2‐63_01142	−1.73	hemC Porphobilinogen deaminase
L2‐63_01143	−1.77	cysG_2 Siroheme synthase, uroporphyrin‐III‐C‐methyltransferase
L2‐63_01144	−1.89	hemB Delta‐aminolevulinic acid dehydratase
L2‐63_01145	−1.39	hemL2 glutamate‐1‐semialdehyde aminotransferase
L2‐63_01146	−2.65	iscR_1 HTH‐type transcriptional regulator
L2‐63_01150	−2.85	trxA_3 Thioredoxin‐M
L2‐63_01151	−3.05	bifunctional sulfur carrier protein
L2‐63_01152	−2.68	moeZ probable adenyltraferase/sulfurtransferase
L2‐63_01153	−2.67	mec CysO‐cysteine peptidase
L2‐63_01154	−2.35	trxB_2 Thioredoxin reductase, alkyl hydroperoxide reductase subunit
L2‐63_01155	−2.09	ifcA fumarate reductase subunit precursor
L2‐63_01156	−2.11	Ferredoxin II, NADH‐plastoquinoine oxidoreductase subunit
L2‐63_01157	−2.13	cysD Sulfate adenylyl transferase subunit 2
L2‐63_01158	−2.52	cysN Sulfate adenylyl transferase subunit 1
**2. Putative branched chain amino acid fermentation gene cluster in *Blautia hydrogenotrophica*, upregulated when in co‐culture with *Ruminococcus bromii***		
RUMHYD_00546	8.27	ilvE branched chain amino acid transaminase
RUMHYD_00547	7.66	branched chain amino acid transport protein
RUMHYD_00548	6.48	brnQ branched chain amino acid transport system II carrier protein
RUMHYD_00549	8.54	2‐oxoacid:acceptor oxidoreductase gamma subunit
RUMHYD_00550	9.28	thiamine pyrophosphate enzyme
RUMHYD_00551	9.34	pyruvate flavodoxin/ferredoxin oxidoreductase, thiamine diP binding
RUMHYD_00552	7.27	4Fe‐4S binding domain protein
RUMHYD_00553	8.74	hypothetical protein
RUMHYD_00554	9.42	buk butyrate kinase ***(presumed acyl kinase)***
RUMHYD_00555	9.46	ptb phosphate butyryltransferase ***(presumed phosphate acyltransferase)***
RUMHYD_00556	1.4	transcriptional regulator, Gnt R family

Transcripts correspond to those within the circles (1) and (2) in Fig. 4 C and D.

This cluster of linked genes also includes six upregulated genes (L2‐63_01140 to L2‐63_01145) encoding proteins involved in porphyrin/siroheme biosynthesis (hemA, cysG_1/MET8, hemC, cysG_2/cobA‐hemD, hemB, hemL) (Table 1, Supporting Information Table S3). Siroheme is a redox‐active cofactor and transcription of genes responsible for its biosynthesis is sensitive to cellular redox potential and/or the redox potential of the fermentation environment (Stroupe *et al*., 2003). Again, with the acetogen potentially providing an extra sink for cellular reductant through a process mediated by hydrogenases, there may be less overall demand for such *R. bromii* proteins mediating redox reactions. Broadly similar changes involving the IscR regulator have been reported in the transcriptome of *Clostridium thermocellum* in response to redox status (Sander *et al*., 2015).

**Figure 5:**
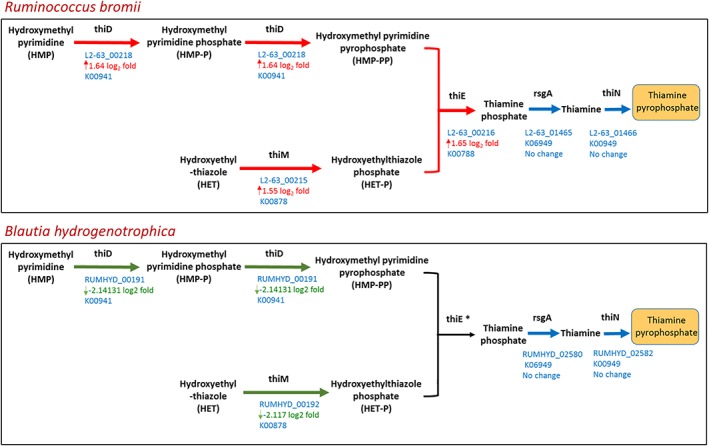
Thiamine salvage pathway in *R. bromii* and *B. hydrogenotrophica*. Changes in expression of genes concerned with thiamine salvage in continuous co‐cultures between *R. bromii* and *B. hydrogenotrophica*. Upregulation is indicated by red arrows (*R. bromii*) and downregulation by green arrows (*B. hydrogenotrophica*). The asterisk indicates no transcript could be identified for thiE in *B. hydrogenotrophica*, but it cannot be ruled out that this activity is encoded by an unknown (hypothetical) protein.

Our data also reveal upregulation of four transcripts associated with thiamine salvage (the thiMDE cluster) and thiamine transport (thiT) in the co‐culture relative to the monoculture (L2‐63_00215 to L2‐63_00216, L2‐63_00218 and L2‐63_01258) (Supporting Information Table S3, Fig. 5). In most organisms, thiamine biosynthesis involves separate synthesis of thiazole and pyrimidine moieties that are coupled to form thiamin phosphate (Rodionov *et al*., 2002; Du *et al*., 2011). *R. bromii* however appears to possess the alternative salvage pathway (thiMDE) (Rodionov *et al*., 2002) (Fig. 5).

### *Changes in the* B. hydrogenotrophica *transcriptome in continuous co‐culture with* R. bromii

A total of 420 *B. hydrogenotrophica* genes showed differences in expression in the co‐culture relative to the monoculture (10.85% of the total genes annotated for *B. hydrogenotrophica* strain) of which 197 were upregulated and 223 were downregulated. One hundred and thirty six of the differentially expressed genes were hypothetical proteins (Supporting Information Table S3). KEGG‐based pathway analysis of the differentially expressed genes in *B. hydrogenotrophica* indicated that multiple pathways were significantly altered in response to co‐culture (enrichment *p* value ≤0.05).

Transcripts in *B. hydrogenotrophica* that showed the highest and most significant upregulation in the co‐culture relative to the monoculture are encoded by a cluster of 11 linked genes (RUMHYD_00546 to RUMHYD_00556) (Table 1, Supporting Information Table S3). These include homologues of butyrate kinase and phosphotransbutyrylase, together with a branched chain fatty acid transporter and a branched chain fatty acid transaminase and a keto acid oxidoreductase. This is in agreement with the observed formation of branched chain fatty acids in the co‐cultures (Fig. 3B). Thus, we propose that the upregulation of these genes enable *B. hydrogenotrophica* to form 2‐oxo acids by transamination that are then converted to branched chain fatty acids with the production of ATP in a manner directly analogous to the conversion of pyruvate to acetate. The two upregulated genes that were computationally annotated as butyrate kinase and phosphate butyryltransferase are predicted to encode branched chain fatty acid kinases and phosphorylases, respectively, since there is no evidence for butyrate production by *B. hydrogenotrophica*.

Another cluster of 7 linked genes (RUMHYD _00315 to RUMHYD _00321) that showed significant upregulation in the co‐culture compared to *B. hydrogenotrophica* monoculture, was concerned with acetogenesis from formate and from CO_2_ (Supporting Information Table S3). KEGG analysis (Supporting Information Fig. S4) also indicated downregulation of several pathways involved in carbohydrate metabolism. This suggests a major shift from dependence on glucose utilization in the monoculture to the utilization of branched chain amino acids and acetogenesis in the co‐culture.

Interestingly, transcripts associated with thiamine biosynthesis (thiC, thiD, thiM, thiW, thiI) were downregulated in *B. hydrogenotrophica* in the co‐culture (Fig. 5) which contrasts with the parallel upregulation of the thiamine biosynthesis transcripts of *R. bromii* (Supporting Information Table S3, Fig. 5). Transcripts involved in biosynthesis of panthothenate and Co‐A were also detected in *B. hydrogenotrophica* (Supporting Information Fig. S5). *B. hydrogenotrophica* possesses most of the enzymes required for the generation of pantothenate (except for pantoate‐beta‐alanine ligase, E6.3.2.1, although there are unannotated hypothetical genes in the current draft genome and one of these may encode this function) and the three transcripts required to produce pantoate from valine were upregulated in the co‐culture (Supporting Information Fig. S5). *R. bromii* does not appear to have panthothenate biosynthesis capacity; as a result, it must rely on the available pantothenate provided in the growth medium, perhaps leading to upregulation of this pathway in *B. hydrogenotrophica*.

## Conclusions

Acetate reaches the highest concentrations among the SCFA produced by anaerobic fermentation in the human large intestine and a ratio of approximately 3:1:1 acetate:propionate:butyrate is typically reported for faecal samples from healthy individuals. Acetate is utilized by many abundant species of butyrate‐producers in the human colon (Barcenilla *et al*., 2000; Louis and Flint, 2017) but its production is widespread among gut anaerobes. Many gut bacteria also produce formate as a major product in pure culture but little or no formate is normally detected in faecal samples. The observations reported here indicate that formate utilization by acetogenic bacteria may be a significant factor explaining the high acetate production and low formate levels that are seen in vivo. Formate is known to be utilized by some methanogenic archaea, but these organisms are present at very low levels in around 50% of the population (Florin *et al*., 2000). Acetogenic bacteria, however, appear to be widespread in the human colon (Rey *et al*., 2010). The Wood‐Ljungdahl pathway found in acetogens was estimated to contribute 30% of total acetate formation in human faecal incubations with ^13^CO_2_ incorporation into acetate exceeding its conversion to methane even in methanogenic individuals (Wolin *et al*., 1999). Interestingly isotope enrichment was found to be less in the methyl group than in the carboxyl group of acetate, which the authors suggested was likely to be due to the contribution of unlabelled formate derived from carbohydrate fermentation (Wolin *et al*., 1999). This is consistent with the type of cross‐feeding observed in the present study. As shown here, *B. hydrogenotrophica* can ferment carbohydrates and can grow ‘mixotrophically’ with net consumption of formate, in addition to being able to utilize H_2_ and CO_2_ to produce acetate (Leclerc *et al*., 1997). The phylogenetic distribution of acetogenesis has yet to be fully established, but it seems likely that a number of human colonic Lachnospiraceae in addition to *Blautia hydrogenotrophica* are able to convert formate into acetate via the Wood‐Ljungdahl pathway (Bernalier *et al*., 1996; Leclerc *et al*., 1997; Wolf *et al*., 2016). Many human colonic acetogens cannot grow with H_2_ and CO_2_ alone (Kamlage *et al*., 1997) and the metabolism of *Bryantella formatexigens* in the presence of fructose, for example, becomes increasingly acetogenic at high formate concentrations (Wolin *et al*., 2003). Recent theoretical models of human gut microbial metabolism are reported to underestimate acetate and overestimate formate concentrations relative to the measured outputs of complex communities studied in vitro (Kettle *et al*., 2015). Our findings suggest that this discrepancy might be resolved by incorporating formate utilization by acetogens into the models.

*Ruminococcus bromii* is a dominant member of the human intestinal microbiota whose population is readily increased by consumption of diets rich in resistant starch when this species can account for more than 10% of total faecal bacteria (Martínez *et al*., 2010; Walker *et al*., 2011). We can conclude that the metabolic consequences of an increased population of *R. bromii* are likely to be to boost acetate production, first through direct formation and second via conversion of formate to acetate through acetogens. In the case of the other major product seen in pure culture, ethanol, it remains to be established whether this is also subject to consumption by other species in the mixed community. It has been suggested that acetogens, such as *B. hydrogenotrophica*, may be favoured under conditions of mildly acidic pH and substrate availability that prevail in the proximal colon (Macfarlane and Gibson, 1997). The proximal colon would therefore be expected to favour this species and also to support high populations of *R. bromii* when fermentable resistant starch is being supplied from the diet. The co‐localisation of these two species seems likely, therefore, but this remains to be established experimentally. Ideally this would be done using techniques that allow in situ sampling of colonic lumen contents containing partially degraded starch particles.

In addition to formate cross‐feeding, our transcriptome analysis has revealed several other significant nutritional interactions that involve amino acid catabolism, electron disposal and vitamin synthesis. Intriguingly, genes concerned with thiamine synthesis were upregulated in *R. bromii* and downregulated in *B. hydrogenotrophica* in the co‐culture. Thiamine is required for the crucial pyruvate ferredoxin oxidoreductase reaction. It is not clear why its synthesis should be upregulated in *R. bromii* in the presence of very low numbers of *B. hydrogenotrophica*, but this may be a consequence of widespread changes in anaerobic metabolism triggered by the acetogen as evidenced by the gene expression changes affecting sulfate metabolism and siroheme in *R. bromii*. The greatly increased expression of genes that we infer to be responsible for branched chain amino acid fermentation by *B. hydrogenotrophica* in the co‐culture is likely to be a response to a limited supply of starch‐derived sugars released by *R. bromii*. In conclusion, this analysis has provided a unique insight into the complex nutritional interplay and interdependence of two of the dominant species that make up a healthy human gut microbiome.

## Experimental procedures

### *Bacterial strains and growth conditions*

*Ruminococcus bromii* L2‐63 is a Rowett isolate described previously (Ze *et al*., 2012). *Blautia hydrogenotrophica* DSM 10507 was purchased from Deutsche Sammlung von Mikrooganismen und Zellkulturen (DSMZ). Cultures were routinely maintained by growing for 16–18 h at 37 °C in 7.5 ml aliquots of anaerobic M2GSC medium (Miyazaki *et al*., 1997) under O_2_‐free CO_2_. Batch culture studies with *R. bromii* and *B. hydrogenotrophica* were carried out in RUM medium (Ze *et al*., 2015) that was simplified here by the omission of supplementary minerals to consist of (per 100 ml): casitone (1.0 g), yeast extract (0.25 g), NaHCO_3_ (0.4 g), cysteine (0.1 g), K_2_HPO_4_ (0.045 g), KH_2_PO_4_ (0.045 g) NaCl, (0.09 g) (NH_4_)_2_SO_4_ (0.09 g), MgSO_4_·7H_2_O (0.009 g), CaCl_2_, (0.009 g) resazurin (0.1 mg), biotin (1 μg), cobalamin (1 μg), *p*‐aminobenzoic acid (3 μg), folic acid (5 μg), pyridoxamine (15 μg). In addition, the following SCFA are included (final concentrations): acetate (33 mM), propionate (9 mM), iso‐butyrate, iso‐valerate and valerate (each 1 mM). Cysteine (0.1 g/100 ml) is added to the medium following boiling and dispensed into Hungate tubes while the tubes are flushed with CO_2_. After autoclaving, filter sterilized solutions of heat labile vitamins were added to give final concentrations of thiamine and riboflavin of 0.05 μg·ml^−1^ (each), pantothenate and nicotinamide of 1 μg·ml^−1^, pantethine of 50 μg·ml^−1^ and tetrahydrofolic acid of 0.1 μg·ml^−1^ as described previously (Ze *et al*., 2015). The final pH of the medium was adjusted to 6.8 ± 0.2. The starch (0.2% final concentration) used for the batch culture incubations was Novelose 330 (resistant starch type 3‐ RS3) which had been boiled for 10 min as described previously (Ze *et al*., 2012) referred to as RUM‐RS (Ze *et al*., 2015) or glucose (0.2%) referred to as RUM‐G.

### *Continuous culture*

Single‐stage fermentor systems, with working volumes of 250 ml were set‐up as described previously (Chung *et al*., 2016) at a constant pH (6.5 ± 0.2) with a continuous flow of the semi defined RUM medium described above with either boiled (100 °C for 10 min) soluble potato starch (Sigma, S2004) or glucose as the carbon source. The fermentor culture vessels were maintained under a stream of CO_2_ at a constant temperature of 37°C using thermal jackets. The medium reservoir and fermentor culture vessel were mixed by internal stirrer bars powered by external stirring units. The volume of the culture was kept constant at 250 ml with a constant flow of fresh medium at a turnover of 250 ml day^−1^ with fresh heat labile vitamins added daily to give final concentrations of thiamine and riboflavin of 0.05 μg·ml^−1^ (each), pantothenate and nicotinamide of 1 μg·ml^−1^, pantethine of 50 μg·ml^−1^ and tetrahydrofolic acid of 0.1 μg·ml^−1^. The pH values of the fermentor vessels were monitored and controlled to 6.5 ± 0.2 using a pH controller which delivers either 0.1 M HCl or 0.1 M NaOH solutions to maintain a constant pH for the full period of the study. Fresh 10 ml cultures of *R. bromii* L2‐63 and *B. hydrogenotrophica* DSM 10507, grown to stationary phase in RUM medium with either soluble potato starch or glucose respectively as energy source, were used to inoculate the fermentors.

### *Total sugar and reducing sugar analysis*

Total sugars were determined by the phenol sulfuric assay as described previously (Dubois *et al*., 1956). Briefly, 0.5 ml of samples or glucose standards were mixed with 0.5 ml of phenol solution (5%, w/v in d.H_2_O), then 2.5 ml of concentrated sulfuric acid (95%–98%) was added to the tubes were allowed to cool, then 200 μl were transferred into 96‐well flat bottom plate and OD_490_ was read using a Safire2 microplate reader (Tecan Group, Männedorf, Switzerland). Reducing sugars were measured separately (Lever, 1977) by transferring 100 μl of culture supernatants or glucose standards into 2.5 ml of 4‐hydroxybenzhydrazide (PAHBAH) reagent. After incubation at 70 °C for 10 min, 200 μl were transferred into 96‐well flat bottom plate and OD_415_ read on a Safire2 microplate reader (Tecan Group, Männedorf, Switzerland).

### *SCFA and ethanol analysis*

SCFA concentrations were measured by gas chromatography as described previously (Richardson *et al*., 1989). Following derivatization of the samples using *N*‐tert‐butyldimethylsilyl‐*N*‐methyltrifluoroacetamide, the samples were analysed using a Hewlett Packard gas chromatograph (GC) fitted with a silica capillary column using helium as the carrier gas. Ethanol concentrations were also measured by gas chromatography using the same GC but fitted with a ZB WAX column.

### *Quantitative PCR*

Real time qPCR was performed in duplicate with iTaq™ Universal SYBR® Green Supermix (Bio‐Rad, Laboratories Ltd, Hertfordshire, UK) in a total volume of 10 μl and 2 ng of DNA in a CFX384TM Real‐time System (Bio‐Rad) as described previously (Chung *et al*., 2016). The primer pairs used are described in Supporting Information Table S4. Annealing temperature was 60 °C for universal and *R. bromii* primers and 63 °C for *B. hydrogenotrophica* primers. Standard curves consisted of 10‐fold dilution series of amplified bacterial 16S rRNA genes from the two strains. Samples were amplified with universal primers against total bacteria and specific primers against *R. bromii* and *B. hydrogenotrophica*. Relative bacterial concentrations in each sample were estimated by comparing the gene copy numbers calculated using standard curves. Data were analysed using BioRad CFX manager software and the detection limit was determined with negative controls containing only herring sperm DNA.

### *RNA extraction*

Total RNA was extracted from 10 ml of culture using phenol‐chloroform extraction followed by ethanol precipitation. Any residual DNA was removed using DNase‐treatment with TURBO DNA‐free kit (Invitrogen). RNA concentration and purity were determined spectrophotometrically using Nano drop Spectrophotometer ND‐1000, BioAnalyzer 2100 (Agilent Technologies UK Ltd, Edinburgh, Scotland, UK) and Qubit (Thermo Fisher Scientific, Perth, Scotland, UK). Ribosomal RNA was depleted in the samples using the Ribo‐Zero rRNA removal kit for bacteria (Illumina, San Diego, CA, USA) according to manufacturer's protocol. mRNA was then purified using RNeasy MinElute kit (Qiagen, Crawley, UK).

### *RNA‐seq*

A total of nine RNA libraries (three replicates each of *R. bromii* monoculture, *B. hydrogenotrophica* monoculture and *R. bromii* – *B. hydrogenotrophica* co‐culture) from the fermenter systems were prepared using the adapted TruSeq RNA protocol (Illumina 15026495 Rev.B). The library preparation involved QC of the RNA (Pico kit, Bioanalyzer 2100, Agilent Technologies) to detect any potential rRNA contamination. The ribo‐depleted RNA was enzymatically fragmented and double strand (ds) cDNA was synthesized using random hexamers in the presence of reverse transcriptase. The samples were end repaired, A‐tailed, adaptor‐ligated, fractionated, purified and enriched. The insert size of the libraries was verified by running an aliquot of the DNA library on a PerkinElmer GX using the High Sensitivity DNA chip (PerkinElmer CLS760672, Perkin Elmer Ltd, Beaconsfield, UK) and the concentration was determined using a High Sensitivity Qubit assay. Libraries were sequenced on an Illumina HiSeq 2500 instrument to generate 100 bp paired end reads. Sequence reads were subjected to post processing to trim Illumina adapters and primer sequences. Sequencing was done at the Earlham Institute (BBSRC Genome Analysis Centre), Norwich, UK.

### *Transcriptome analysis*

High quality reads for each sample were aligned against the combined reference genomes of *R. bromii* L2‐63 and *B. hydrogenotrophica* DSM 10507, downloaded from GenBank repository (GCA_000209875.1 and GCA_000157975.1). Associated annotation file in gff3 format was used to obtain information for downstream analysis. Alignments were generated using TopHat (version 2.1.0 release 6/29/2015) with the ‐‐max‐multihits 1 option (Trapnell *et al*., 2009). Read counts were generated using HTSeq‐count (version 0.6.1) (Anders *et al*., 2015). Gene expression for each sample was computed as a measure of the total number of reads uniquely aligning to the reference, binned by genic coordinates (information acquired from the annotation file). Differential gene expression analysis was performed using the Bioconductor package DESeq2 (version 1.14.0) (Anders and Huber, 2010). Raw read counts thus obtained were normalized to account for differences in sequencing depth and composition using methods implemented within DESeq. Differential expression of pairwise comparisons (of the different groups) was assessed using the negative binomial test with Benjamani–Hochberg false discovery rate (FDR) adjustment (Hochberg and Benjamini, 1990) applied for multiple testing corrections. For this study, an FDR of 0.05 was applied and any candidate that had a *p*‐adjusted value of ≤ 0.05 was considered to be significantly up‐ or downregulated. To gain further insights into the effects of co‐cultivation, we performed a KEGG pathway analysis. KEGG orthology was assigned to all *R. bromii* and *B. hydrogenotrophica* genes using KAAS‐KEGG (Moriya *et al*., 2007). KAAS‐KEGG outputs were then converted to GMT format and combined with KEGG pathway information gathered using the KEGG Rest API (http://www.kegg.jp/kegg/rest/). Analysis of significant enrichment of KEGG pathways in the differentially expressed gene lists was then carried out using the GO Enrichment function of the software package Partek® Genomics Suite® software version 6.6 Copyright ©; 2016. The Fisher Exact test function was used only analysing pathways with more than two genes present and pathways with a *p* < 0.05 were deemed to be significantly enriched.

### *Data availability*

RNA‐seq data have been deposited in the ArrayExpress database at EMBL‐EBI (www.ebi.ac.uk/arrayexpress) under accession number E‐MTAB‐ 210350.

## Author contributions

HJF devised the study. JLG, IM, SD performed the experiments. JLG, IM, HJF, PL, SD, SS and EDG analysed the data and prepared figures and tables. EC and NJ provided critical resources and support. HJF, IM, SD wrote the paper. All authors read and approved the final manuscript.

## Supporting information


**Fig. S1.** Effect of formate on growth and metabolism of *Blautia hydrogenotrophica* in RUM medium. a) shows growth (OD_650_) with or without the addition of 10 mM formate and/or 0.2% (11 mM) glucose to the basal medium; b) and c) Metabolites formed and utilized during growth on glucose with or without formate (for the experiment shown in a). Values represent means of triplicate cultures.

**Fig. S2.** Growth (OD_650_) of *Ruminococcus bromii*, *Blautia hydrogenotrophica* and co‐cultures in continuous culture. Results are shown for six fermentors. Vessels R1 and C1 were inoculated simultaneously with *R. bromii* only (R1) or with both bacteria (C1) and supplied with RUM‐S medium containing 0.5% soluble starch. R2 and C2 refer to a repeat of this experiment. B1 and B2 vessels were run separately and supplied with RUM‐G medium containing 0.5% glucose (B1) or 0.2% glucose (B2); these vessels were inoculated with *B. hydrogenotrophica* only.

**Fig. S3.** Metabolite concentrations for the six fermentor experiments shown in Fig. S2**.** Blue arrows indicate the sampling points used for RNAseq analysis.

**Fig. S4.** KEGG Carbon metabolism pathway map for *Blautia hydrogenotrophica*. Genes present in the differentially expressed gene (DEG) list are highlighted with those with up regulated expression in the co‐culture compared to the mono‐culture in red and those with down regulated expression in green. Genes with KEGG orthologs found by KAAS‐KEGG, but no significant change in expression, are highlighted in blue. Numbers inside boxes are enzyme commission numbers.

**Fig. S5.** KEGG pathway map for metabolism of pantothenate and CoA in *Blautia hydrogenotrophica*. Genes present in the differentially expressed gene (DEG) list are highlighted with those with up regulated expression in the co‐culture compared to the mono‐culture in red and those with down regulated expression in green. Genes with KEGG orthologs found by KAAS‐KEGG, but no significant change in expression, are highlighted in blue.

**Table S1.** Calculated carbon balances for monocultures and co‐cultures**.**

**Table S2.** RNAseq read counts.

**Table S3.** Differentially expressed genes (DEGs) list from *Ruminococcus bromii* and *Blautia hydrogenotrophica*.

**Table S4.** Primers for qPCR.
