# Supplementary material for: Formate cross‐feeding and cooperative metabolic interactions revealed by transcriptomics in co‐cultures of acetogenic and amylolytic human colonic bacteria
Source: Environ Microbiol. 2018 Nov 22;21(1):259–71. doi: 10.1111/1462-2920.14454 (PMC6378601; doi:10.1111/1462-2920.14454)
Supplement: Supplementary file 1 — Fig. S1. Effect of formate on growth and metabolism of Blautia hydrogenotrophica in RUM medium. a) shows growth (OD650) with or without the addition of 10 mM formate and/or 0.2% (11 mM) glucose to the basal medium; b) and c) Metabolites formed and utilized during growth on glucose with or without formate (for the experiment shown in a). Values represent means of triplicate cultures. [file EMI-21-259-s001.pptx]

## Slide 1
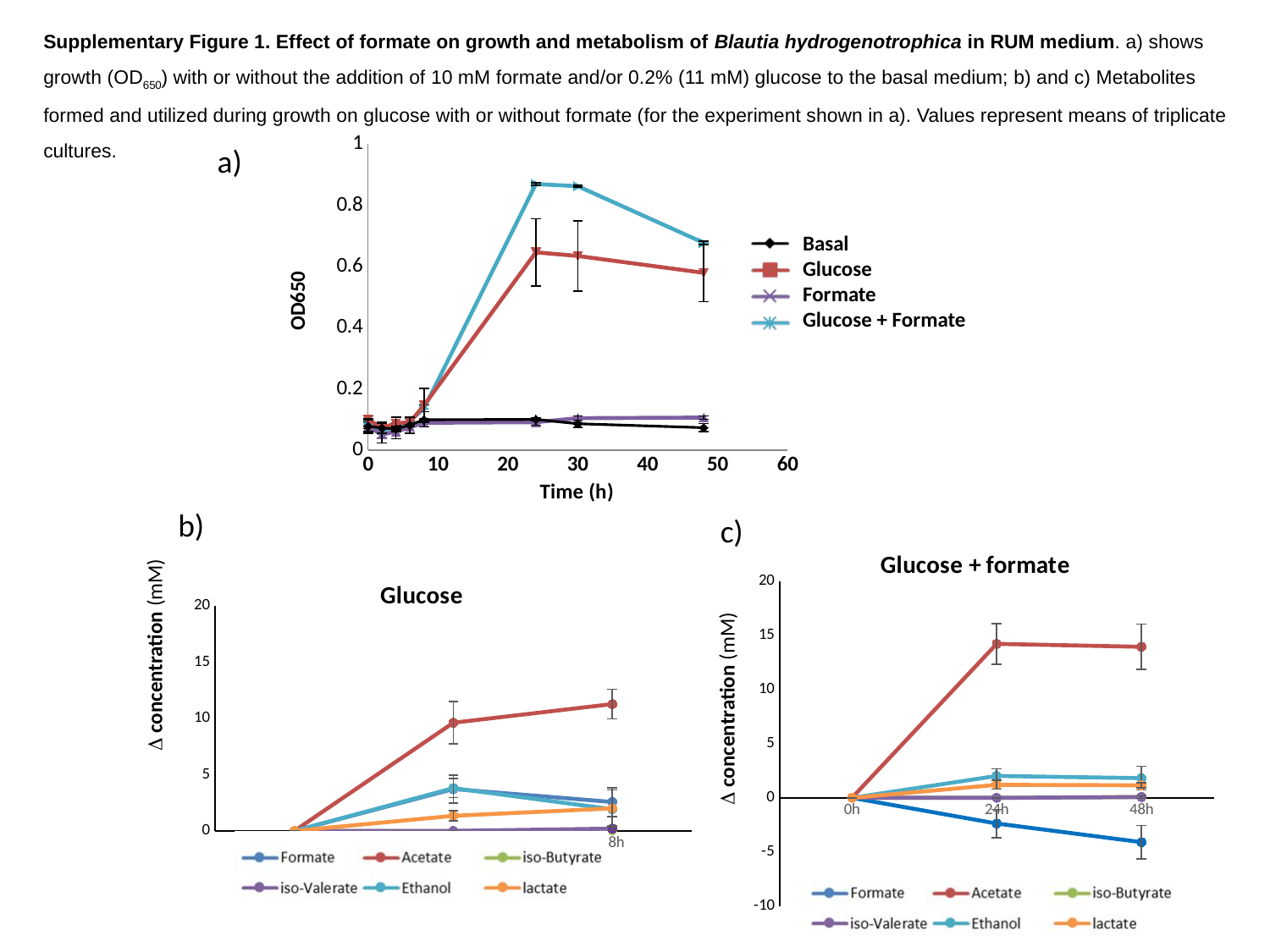

Supplementary Figure 1. Effect of formate on growth and metabolism of Blautia hydrogenotrophica in RUM medium. a) shows growth (OD650) with or without the addition of 10 mM formate and/or 0.2% (11 mM) glucose to the basal medium; b) and c) Metabolites formed and utilized during growth on glucose with or without formate (for the experiment shown in a). Values represent means of triplicate cultures.
### Chart
| Category | Basal | 0.2% glc | 10 mM form | glc + form |
|---|---|---|---|---|a)
Basal
Glucose
Formate
Glucose + Formate
### Chart: Glucose + formate
| Category | Formate | Acetate | Iso-B | Iso-V | Ethanol | lactate |
|---|---|---|---|---|---|---|
| 0h | 0.0 | 0.0 | 0.0 | 0.0 | 0.0 | 0.0 |
| 24h | -2.37 | 14.22 | 0.0 | 0.0 | 2.02 | 1.2 |
| 48h | -4.09 | 13.95 | 0.08 | 0.07 | 1.82 | 1.16 |b)
c)
### Chart: Glucose
| Category | Formate | Acetate | Iso-B | Iso-V | Ethanol | lactate |
|---|---|---|---|---|---|---|
| 0h | 0.0 | 0.0 | 0.0 | 0.0 | 0.0 | 0.0 |
| 24h | 3.72 | 9.61 | 0.0 | 0.0 | 3.8 | 1.34 |
| 48h | 2.57 | 11.27 | 0.07 | 0.2 | 1.92 | 2.0 |D concentration (mM)
D concentration (mM)
