# Supplementary material for: Formate cross‐feeding and cooperative metabolic interactions revealed by transcriptomics in co‐cultures of acetogenic and amylolytic human colonic bacteria
Source: Environ Microbiol. 2018 Nov 22;21(1):259–71. doi: 10.1111/1462-2920.14454 (PMC6378601; doi:10.1111/1462-2920.14454)
Supplement: Supplementary file 2 — Fig. S2. Growth (OD650) of Ruminococcus bromii, Blautia hydrogenotrophica and co‐cultures in continuous culture. Results are shown for six fermentors. Vessels R1 and C1 were inoculated simultaneously with R. bromii only (R1) or with both bacteria (C1) and supplied with RUM‐S medium containing 0.5% soluble starch. R2 and C2 refer to a repeat of this experiment. B1 and B2 vessels were run separately and supplied with RUM‐G medium containing 0.5% glucose (B1) or 0.2% glucose (B2); these vessels were inoculated with B. hydrogenotrophica only. [file EMI-21-259-s002.pptx]

## Slide 1
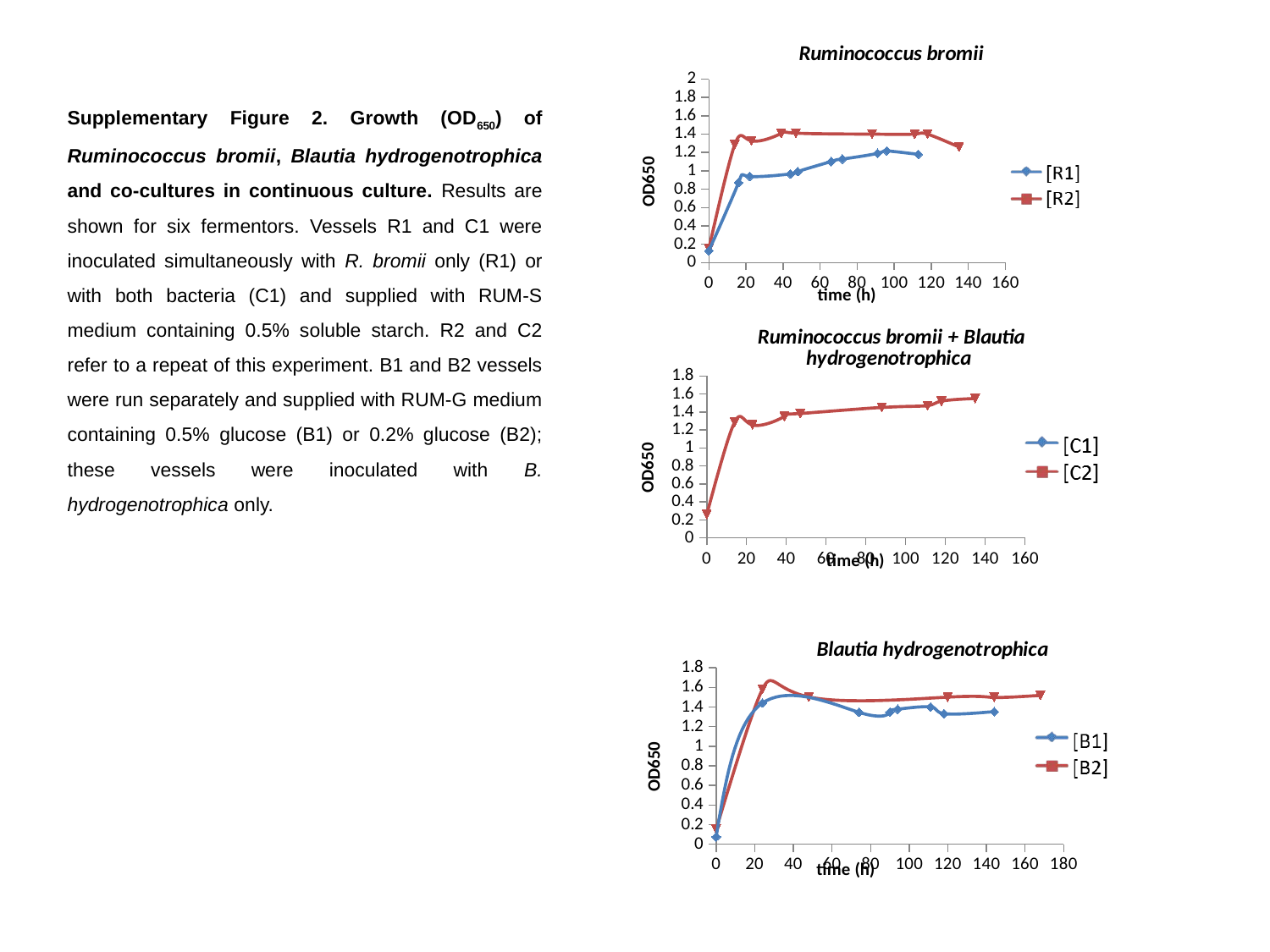

### Chart: Ruminococcus bromii
| Category | 22/06/2015 | 01/07/2015 |
|---|---|---|
### Chart: Ruminococcus bromii + Blautia hydrogenotrophica
| Category | 22/06/2015 | 01/07/2015 |
|---|---|---|
### Chart: Blautia hydrogenotrophica
| Category | 09/07/2015 | 11/11/2015 |
|---|---|---|Supplementary Figure 2. Growth (OD650) of Ruminococcus bromii, Blautia hydrogenotrophica and co-cultures in continuous culture. Results are shown for six fermentors. Vessels R1 and C1 were inoculated simultaneously with R. bromii only (R1) or with both bacteria (C1) and supplied with RUM-S medium containing 0.5% soluble starch. R2 and C2 refer to a repeat of this experiment. B1 and B2 vessels were run separately and supplied with RUM-G medium containing 0.5% glucose (B1) or 0.2% glucose (B2); these vessels were inoculated with B. hydrogenotrophica only.
