# Supplementary material for: Formate cross‐feeding and cooperative metabolic interactions revealed by transcriptomics in co‐cultures of acetogenic and amylolytic human colonic bacteria
Source: Environ Microbiol. 2018 Nov 22;21(1):259–71. doi: 10.1111/1462-2920.14454 (PMC6378601; doi:10.1111/1462-2920.14454)
Supplement: Supplementary file 3 — Fig. S3. Metabolite concentrations for the six fermentor experiments shown in Fig. S2. Blue arrows indicate the sampling points used for RNAseq analysis. [file EMI-21-259-s003.pptx]

## Slide 1
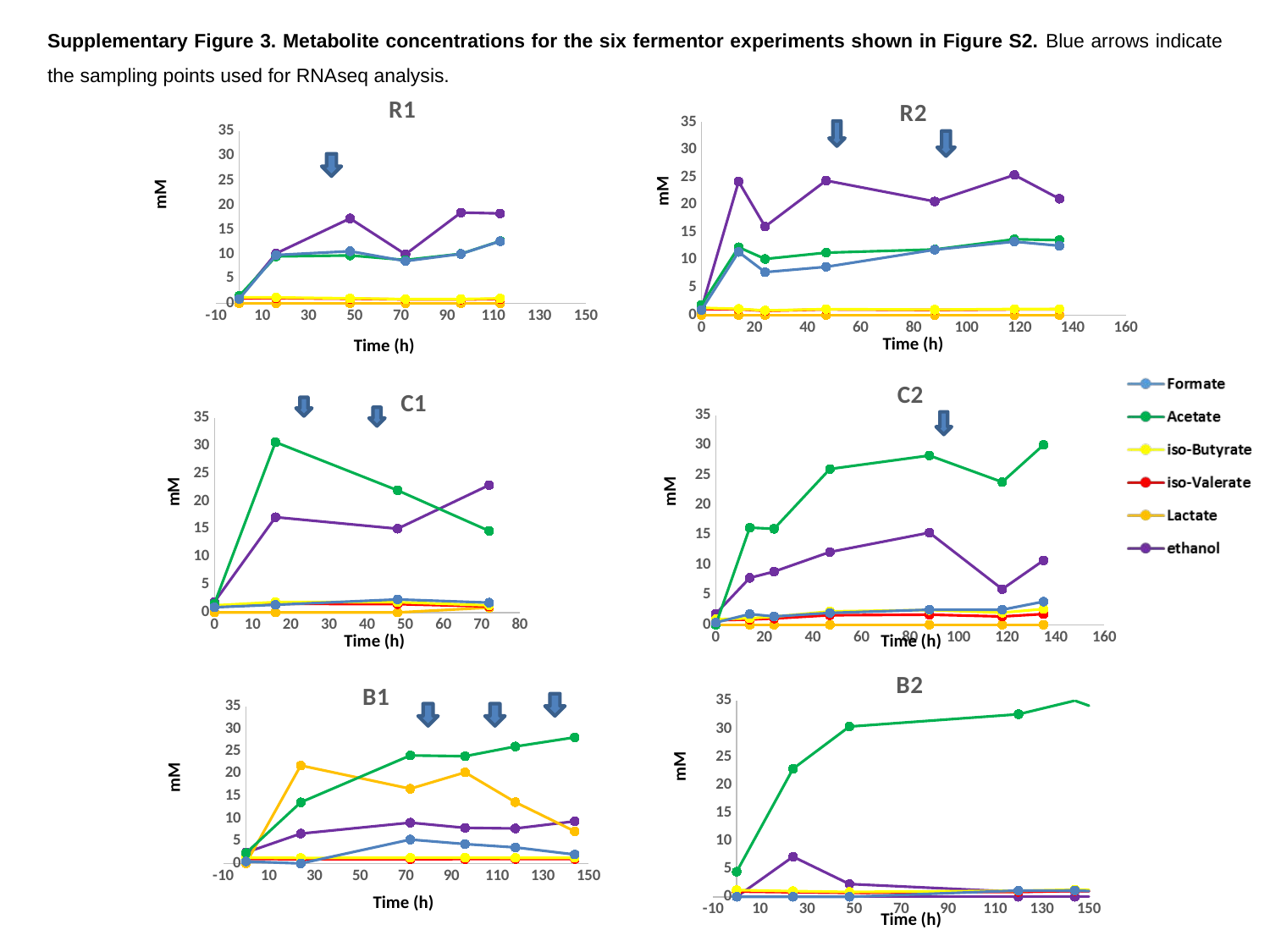

Supplementary Figure 3. Metabolite concentrations for the six fermentor experiments shown in Figure S2. Blue arrows indicate the sampling points used for RNAseq analysis.
### Chart: R1
| Category | Formate | Acetate | iso-Butyrate | iso-Valerate | Lactate | ethanol |
|---|---|---|---|---|---|---|
### Chart: R2
| Category | Formate | Acetate | iso-Butyrate | iso-Valerate | Lactate | ethanol |
|---|---|---|---|---|---|---|
mM
mM
Time (h)
Time (h)
### Chart: C2
| Category | Formate | Acetate | iso-Butyrate | iso-Valerate | Lactate | ethanol |
|---|---|---|---|---|---|---|
### Chart: C1
| Category | Formate | Acetate | iso-Butyrate | iso-Valerate | Lactate | ethanol |
|---|---|---|---|---|---|---|
mM
mM
Time (h)
Time (h)
### Chart: B2
| Category | Formate | Acetate | iso-Butyrate | iso-Valerate | Lactate | ethanol |
|---|---|---|---|---|---|---|
### Chart: B1
| Category | Formate | Acetate | iso-Butyrate | iso-Valerate | Lactate | ethanol |
|---|---|---|---|---|---|---|
mM
mM
Time (h)
Time (h)
