# Supplementary material for: Formate cross‐feeding and cooperative metabolic interactions revealed by transcriptomics in co‐cultures of acetogenic and amylolytic human colonic bacteria
Source: Environ Microbiol. 2018 Nov 22;21(1):259–71. doi: 10.1111/1462-2920.14454 (PMC6378601; doi:10.1111/1462-2920.14454)
Supplement: Supplementary file 4 — Fig. S4. KEGG Carbon metabolism pathway map for Blautia hydrogenotrophica. Genes present in the differentially expressed gene (DEG) list are highlighted with those with up regulated expression in the co‐culture compared to the mono‐culture in red and those with down regulated expression in green. Genes with KEGG orthologs found by KAAS‐KEGG, but no significant change in expression, are highlighted in blue. Numbers inside boxes are enzyme commission numbers. [file EMI-21-259-s004.pptx]

## Slide 1
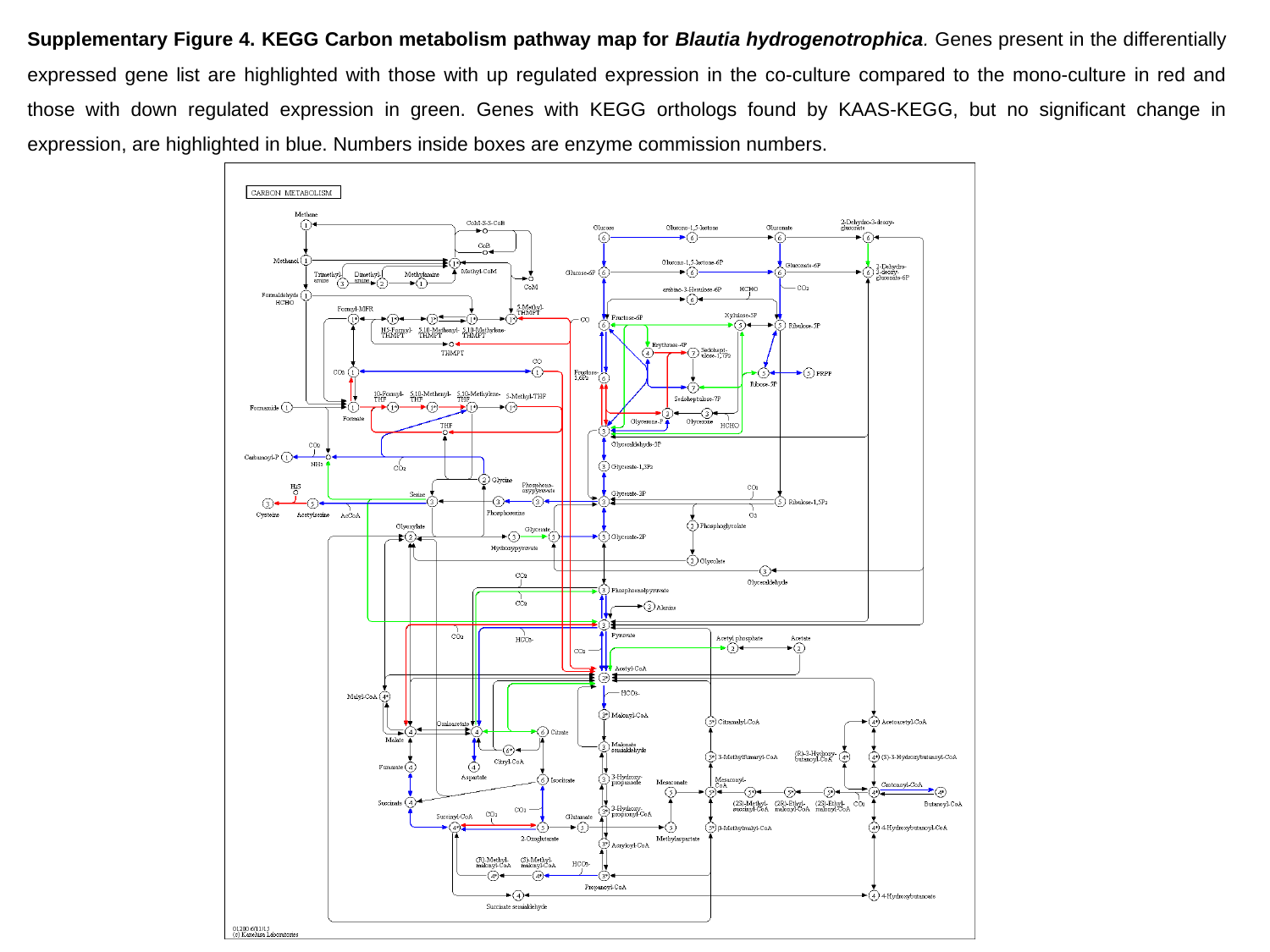

Supplementary Figure 4. KEGG Carbon metabolism pathway map for Blautia hydrogenotrophica. Genes present in the differentially expressed gene list are highlighted with those with up regulated expression in the co-culture compared to the mono-culture in red and those with down regulated expression in green. Genes with KEGG orthologs found by KAAS-KEGG, but no significant change in expression, are highlighted in blue. Numbers inside boxes are enzyme commission numbers.
