# Supplementary material for: Formate cross‐feeding and cooperative metabolic interactions revealed by transcriptomics in co‐cultures of acetogenic and amylolytic human colonic bacteria
Source: Environ Microbiol. 2018 Nov 22;21(1):259–71. doi: 10.1111/1462-2920.14454 (PMC6378601; doi:10.1111/1462-2920.14454)
Supplement: Supplementary file 6 — Table S1. Calculated carbon balances for monocultures and co‐cultures. [file EMI-21-259-s006.docx]

**Supplementary Table 1.** Calculated carbon balances for monocultures and co-cultures**.**

***Ruminococcus bromii Co-culture R. bromii Blautia hydro-***

***+ B. hydrogenotrophica genotrophica***

*Batch culture Continuous Batch culture Continuous Continuous*

*culture culture culture ^c^*

mM C2 units mM C2 units mM C2 units mM C2 units mM C2 units

***Consumed:*** *(54 hours) (over 24 hours) (54 hours) (over 24 hours) (over 24 hours)*

Glucose 8.44 25.32 22.03 66.08 10.43 31.29 20.88 62.65 24.54 73.61

***Produced:***

Formate 6.92 3.46 11.63 5.81 0 0 2.71 1.36 0 0

Acetate 6.89 6.89 12.64 12.64 20.47 20.47 27.04 27.04 32 32

Ethanol 7.43 7.43 22.89 22.89 5.87 5.87 11.05 11.05 0 0

Lactate 0 0 0 0 0.69 1.04 0 0 0 0

CO_2_ 4.22^a^ 11.04^a^ 7.46^a^  14.93^a^ 24.54^a^

Sum 22 52.4 34.84 54.38 56.54

C recovery ^d^ 86.9 % 79.3 % 111.3 % 86.8 % 76.81 %

|  |  |
| --- | --- |

1. CO_2_ values given here are estimated on the assumption of 1 mol CO_2_ released per mol glucose utilized (*R. bromii* monoculture), 2 mol/ glucose (*B. hydrogenotrophica* monoculture) and 1.43 mol/ glucose (co-culture) (based on the stoichiometries suggested in Figure 4).
2. Values for batch cultures are from the time course experiment shown in Figure 2. Values for continuous culture are from the second set of experimental runs (R2, C2, B2) using mean metabolite values to calculate production (see Methods).
3. Run B2 (shown here) was supplied with 0.2% glucose whereas run B1 was supplied with 0.5% glucose, which led to @ 25% of carbon appearing as lactate (see …)
4. Carbon recovery refers only to carbohydrate. Additional carbon contributed by fermentation of BCAA was not measured and the products are of BCAA fermentation are not included here (thus the figures for CO_2_ are calculated only for carbohydrate).
