# Supplementary material for: Formate cross‐feeding and cooperative metabolic interactions revealed by transcriptomics in co‐cultures of acetogenic and amylolytic human colonic bacteria
Source: Environ Microbiol. 2018 Nov 22;21(1):259–71. doi: 10.1111/1462-2920.14454 (PMC6378601; doi:10.1111/1462-2920.14454)
Supplement: Supplementary file 7 — Table S2. RNAseq read counts. [file EMI-21-259-s007.docx]

| **Supplementary Table 2.** RNAseq read counts. | |  | |  |
| --- | --- | --- | --- | --- |
|  |  |  | |  |
| **Sample** | **Library** | **Reads** | | **Overall read mapping rate** |
| Rb-A | LIB21775_LDI19072_CCGTCC | 21872304 | | 92.00% |
| Rb-C | LIB21776_LDI19073_GTAGAG | 24075843 | | 90.20% |
| Rb-D | LIB21777_LDI19074_GTCCGC | 24732617 | | 91.30% |
| Rb+Bh-A | LIB21778_LDI19075_GTGAAA | 26271264 | | 89.00% |
| Rb+Bh-B | LIB21779_LDI19076_GTGGCC | 29940453 | | 92.70% |
| Rb+Bh-D | LIB21780_LDI19077_GTTTCG | 28830866 | | 91.50% |
| Bh-A | LIB21781_LDI19078_CGTACG | 27367272 | | 91.40% |
| Bh-B | LIB21782_LDI19079_GAGTGG | 26068139 | | 92.70% |
| Bh-D | LIB21783_LDI19080_GGTAGC | 26316237 | | 92.70% |
| Total reads |  | 235474995 | |  |
| Average |  | 26163888 | |  |
|  |  |  | |  |
| Rb | *Ruminococcus bromii* L2-63 | |  |  |
| Bh | *Blautia hydrogenotrophica* DSM 10507 | |  |  |
