# Supplementary material for: Formate cross‐feeding and cooperative metabolic interactions revealed by transcriptomics in co‐cultures of acetogenic and amylolytic human colonic bacteria
Source: Environ Microbiol. 2018 Nov 22;21(1):259–71. doi: 10.1111/1462-2920.14454 (PMC6378601; doi:10.1111/1462-2920.14454)
Supplement: Supplementary file 9 — Table S4. Primers for qPCR. [file EMI-21-259-s009.docx]

| **Supplementary Table 4.** Primers for qPCR. | | |  |
| --- | --- | --- | --- |
| **Primer name** | **Primer sequence** | **Target group/ specificity** | **Reference** |
| UniF | GTGSTGCAYGGYYGTCGTCA | Universal | Fuller et al, Brit J Nutr 2007 |
| UniR | ACGTCRTCCMCNCCTTCCTC | Universal | Fuller et al, Brit J Nutr 2007 |
| Rflbr730F | GGCGGCYTRCTGGGCTTT | *R. bromii* | Salonen et al, ISME J 2014 |
| RbromR | CAACTTTCCCCGAAGGGCACCTA | *R. bromii* | Salonen et al, ISME J 2014 |
| BlautiaF2 | CGCGTGAAGGAAGAAGTATC | *Blautia spp.* | Vollmer et al, Food Res Int 2017 |
| BlautiaR2 | GAGCCTCAACGTCAGTTACC | *Blautia spp.* | Vollmer et al, Food Res Int 2017 |
